# Dielectric Properties of Epoxy Resin Impregnated Nano-SiO_2_ Modified Insulating Paper

**DOI:** 10.3390/polym11030393

**Published:** 2019-02-28

**Authors:** Qingguo Chen, Hongda Yang, Xinyu Wang, Heqian Liu, Kai Zhou, Xin Ning

**Affiliations:** 1 Heilongjiang Provincial Key Laboratory of Dielectric Engineering, School of Electrical and Electronic Engineering, Harbin University of Science and Technology, Harbin 150080, China; yanghongda_phd16@hrbust.edu.cn (H.Y.); wxy@hrbust.edu.cn (X.W.); zk_hrb@163.com (K.Z.); 18845152252@163.com (X.N.); 2 Key Laboratory of Engineering Dielectrics and Its Application, Ministry of Education, Harbin University of Science and Technology, Harbin 150080, China; 3 State Grid Heilongjiang Electric Power Company Limited Electric Power Research Institute, Harbin 150030, China; heqian_liu@163.com

**Keywords:** dry bushing, epoxy resin-impregnated paper, dielectric characteristics, thermally stimulated depolarization current, nanocomposite

## Abstract

Epoxy resin-impregnated insulation paper (RIP) composites are used as the inner insulation of dry condenser bushing in the ultra-high voltage direct current (UHVDC) power transmission system. To improve the dielectric properties of RIP, nano-SiO_2_ is added to the insulation paper at concentrations of 0–4wt % before impregnation with pure epoxy resin. X-ray diffraction (XRD), scanning electron microscopy observations as well as the typical dielectric properties of relative permittivity, DC volume conductivity, DC breakdown strength, and thermally stimulated depolarization current (TSDC), were obtained. The effects of trap parameters on the breakdown field strength and volume conductivity were investigated. The DC breakdown electric field strength of the sample increased as the trap level increased. The maximum DC breakdown strength of nano-SiO_2_-modified RIP was increased by 10.6% the nano-SiO_2_ content of 2 wt %. The relative permittivity and DC volume conductivity were first decreased and then increased with increasing nano-SiO_2_ content. These changes occurred near the interfaces between nano-SiO_2_ and RIP. The increased DC breakdown strength was mainly attributed to the increased trap level.

## 1. Introduction

**Figure 1:**
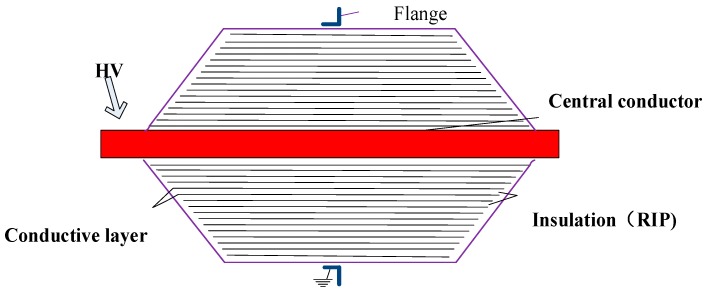
Schematic representation of the condenser core for dry bushing.

Direct-current (DC) transmission systems have smaller line losses and can be more economical in long-distance transmission than alternating-current (AC) transmission systems; the demand for long-distance transmission such as new energy grid-connected, offshore wind power and trans-sea transmission. Therefore, DC transmissions have been vigorously developed worldwide. High voltage is required as transmission power increases. High-voltage DC bushings are key components for DC transmission systems. Dry DC bushings provide excellent mechanical and thermal resistance and avoid flammability problems of oil-impregnated paper bushings. Moreover, the installation angle is more flexible than that of oil-immersed paper insulating bushings. The capacitor core of a high-voltage dry DC bushing is made by alternately rolling a multilayered crepe paper and aluminum foil around the center conductor, vacuum-impregnating the paper with epoxy resin and applying a staged curing process. The main insulation is provided by epoxy resin-impregnated paper (RIP). The structure of a bushing is shown in Figure 1 [1]. However, the manufacturing technology of ultrahigh-voltage DC (UHVDC) bushing has been monopolized by a few companies. At present, only ABB and Siemens have mastered the relevant key manufacturing technologies. However, as the voltage level increases, the weight and length of the bushing increase significantly. A ±1100 kV wall bushing is approximately 25 m long and 18 tons in weight. Enhancing the insulation properties could significantly reduce equipment cost and volume while facilitating transportation and installation. Few reports have investigated improving the main insulating properties of UHVDC bushing. Peng et al. fabricated epoxy resin/crepe paper composites and studied their typical dielectric properties, such as relative dielectric constant, dielectric loss factor, space charge characteristics, DC volume resistivity, and breakdown performance [2,3]. These studies provide some experimental basis for the design and manufacture of UHVDC dry bushing.

To improve the properties of dielectrics, many scholars have modified dielectrics materials with corresponding nanoscale or microscale fillers. Epoxy resin has been modified with nanoscale or microscale particles in order to improve the space charge, partial discharge resistance, breakdown strength, and resistivity [4,5,6]. Katayama et al. use nano-SiO_2_ and micron-SiO_2_ to improve the discharge resistance and inhibits pace charge interactions [7]. Lee et al. studied the effect of nano-silver on the electrical properties of epoxy resin [8]. Nezhad et al. studied the effect of nano-carbon content on the quasi-static mechanical performance of epoxy resin [9]. Fillers increase the viscosity of epoxy resins, affecting their performance in impregnation. Therefore, nanofiller-modified epoxy resin is not suitable for RIP composites. For the oil-impregnated paper composite insulation systems, many scholars have used nano- or microparticles to modify the insulation board to improve the dielectric properties [10]. Kamata et al. used nano-montmorillonite (MMT) and nano-SiO_2_ to reduce the relative dielectric constant of oil-immersed insulating paperboard [11,12,13]. Liao et al. found that nano-TiO_2_ and nano-AlN improved the AC breakdown performance of oil-immersed insulated cardboard, while nano-AlN, ZnO, and TiO_2_ inhibited the space-charge accumulation characteristics of oil-immersed insulated cardboard. [14,15,16,17]. Chen et al. found that nano-SiC particles induced obvious nonlinearity in the DC conductivity and DC electric field strength of oil-immersed insulated pressboard and that nano-Al_2_O_3_ particles enhanced the AC and DC breakdown field strengths of oil-immersed insulated cardboard [18,19]. However, few studies have investigated nano-modified RIP composites.

In order to improve insulation performance of RIP, in this study, pure epoxy resin-impregnated nano-SiO_2_ modified insulating pressboard was fabricated and its main insulation dielectric properties were investigated. The typical dielectric properties of RIP were tested, focusing on the effects of trap parameters on DC breakdown and DC conductivity. Here, the term “trap” refers to a localized state in the forbidden band that constrains ion transfer. Traps are formed by not only the branches and end groups of cellulose, the main component of paper, but also by the lattice defects of nanoparticles in the modified pressboard system [20].

## 2. Materials and Methods 

### 2.1. Sample Preparation

**Figure 2:**
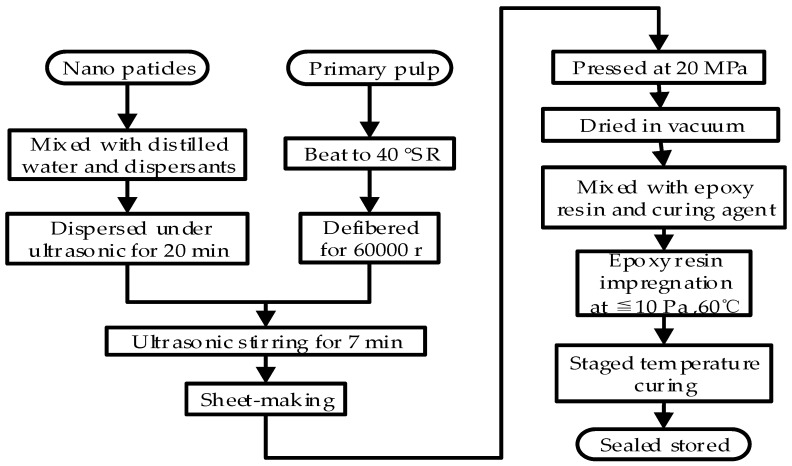
The flow chart of the making process of epoxy resin impregnated nano-SiO_2_ modified pressboard.

The nano-SiO_2_ modified pressboards were composed of unbleached coniferous kraft pulp, distilled water (μ < 10 S/cm), SiO_2_ nanoparticles (Hydrophilic-150) with a diameter of 7–40 nm, purchased from Aladdin Biochemical Technology Co., Ltd. (Shanghai, China). Epoxy resin and curing agent for impregnation are the WSR618 (E-51) matrix purchased from Xingchen Synthetic Material Co., Ltd. (Nantong, China) and methyl hexahydrophthalic anhydride (MHHPA) was bought from Huicheng Electronic Materials Co., Ltd. (Puyang, China). 2,4,6-Tri(dimethylaminomethyl)phenol (DMP-30, Shanfeng Chemical Co., Ltd., Changzhou, China) was used as an accelerant. According to the industrial manufacturing process of insulation pressboards, the samples were made through the seven steps of pulping, doping (with 0, 0.5, 1, 2, 3 or 4 wt % nano-SiO_2_), shaping, compressing, drying, impregnating with epoxy resin and staged temperature curing. These processes used a beater, ultrasonic dispersion instrument, standard agitator, handsheet former, curing press, and vacuum drying chamber, as shown in Figure 2, in which SR is the unit of beating degree.

Polyethylene glycol (PEG, Tianjin Guangfu Chemical Research Institute, Tianjin, China) was used as a modifier to avoid aggregation of nano-SiO_2_. The long-chain structure of PEG has a location-obstructing effect that can prevent the aggregation of nanoparticles in suspension [21]. Moreover, the fine combination with cellulose and retention of nano-SiO_2_ particles was guaranteed by the twining effect from the long-chain structure of PEG [22]. Finally, the epoxy resin impregnated nano-SiO_2_ modified pressboard is obtained with a thickness of 0.25 mm.

**Figure 3:**
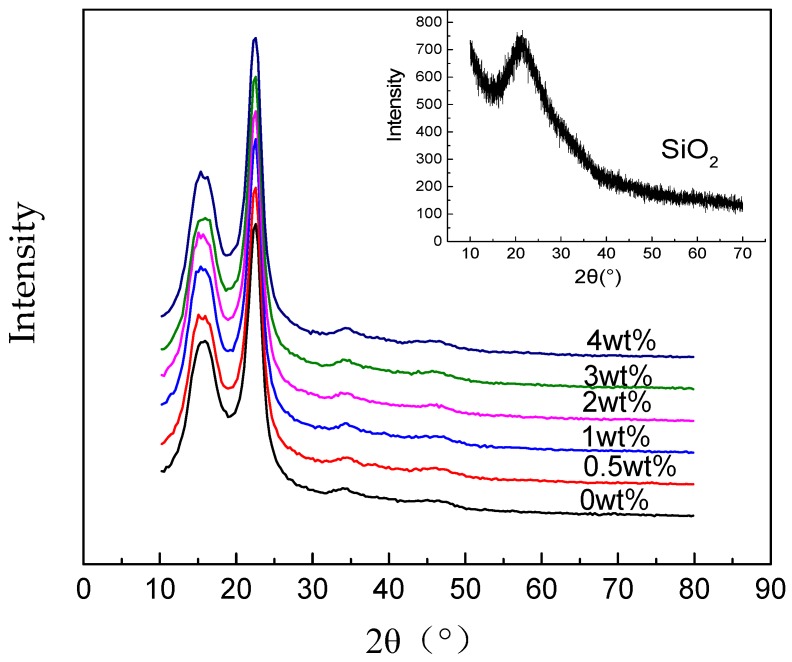
XRD (X-ray diffraction) spectra of non-modified and nano-SiO_2_ modified RIP (epoxy resin-impregnated insulation paper).

The X-ray diffraction (XRD) curves of non-modified and nano-SiO_2_ modified RIP are shown in Figure 3.

In Figure 3, every curve has two peaks while the position and the shape of the characteristic peak are unchanged with the SiO_2_ contents increasing, which implies that the SiO_2_ does not change the main structure of the RIP. However, the SiO_2_ is amorphous and shows no strong characteristic peak, the intensity of SiO_2_ is much smaller than the RIP. Therefore, there is no new peak appears in the modified RIP curves.

**Figure 4:**
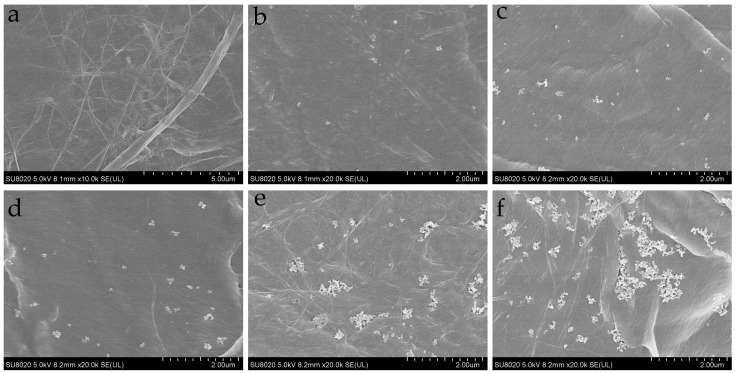
(**a**) SEM (scanning electron microscopy) micrographs of unmodified pressboard; (**b**) SEM micrographs of modified pressboard with 0.5 wt % SiO_2_; (**c**) SEM micrographs of modified pressboard with 1 wt % SiO_2_; (**d**) SEM micrographs of modified pressboard with 2 wt % SiO_2_; (**e**) SEM micrographs of modified pressboard with 3 wt % SiO_2_; (**f**) SEM micrographs of not modified pressboard wit 4 wt % SiO_2._

The microstructures of nano-SiO_2_ modified and unmodified impregnated pressboards are shown in SEM micrographs of Figure 4.

The nano-SiO_2_ particles distribute uniformly in the pressboard when the nano-SiO_2_ content is low. However, with the increasing of the nano-SiO_2_ content the nano-SiO_2_ particles beginning to agglomerate.

### 2.2. Measurement System

**Figure 5:**
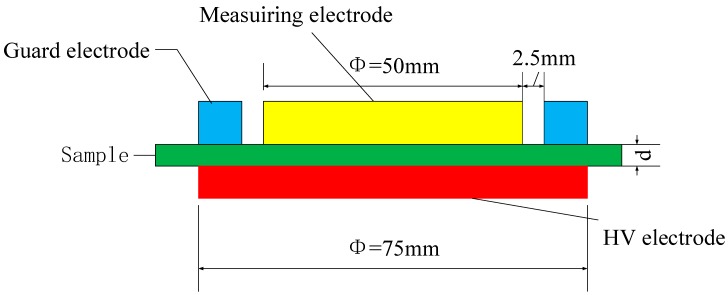
Configuration of the electrode system for conduction current measurement. d is the thickness of the sample.

The DC conductivity characteristics of non-modified and nano-SiO_2_ modified RIP were studied at room temperature by measuring the leakage current with a three-terminal electrode system placed in an oven, as shown in Figure 5. The system was connected to a 6517A electrometer. And an electrical field of 2–30 kV/mm was applied to the sample by DC high-voltage generators. Aluminum was evaporated onto the surface of the sample as electrodes. The stable current (*I*) was recorded after applying the DC voltage for 10 min. For accuracy, the average values of four samples were employed to ensure repeatability.

The relative permittivity of non-modified and nano-SiO_2_-modified pressboard RIP within 10^−1^ to 10^7^ Hz is measured by the Novocontrol broadband dielectric spectrometer with gold-platedcopper electrodes of 20 mm in diameter. Aluminum was evaporated onto the surface of the sample as electrodes.

**Figure 6:**
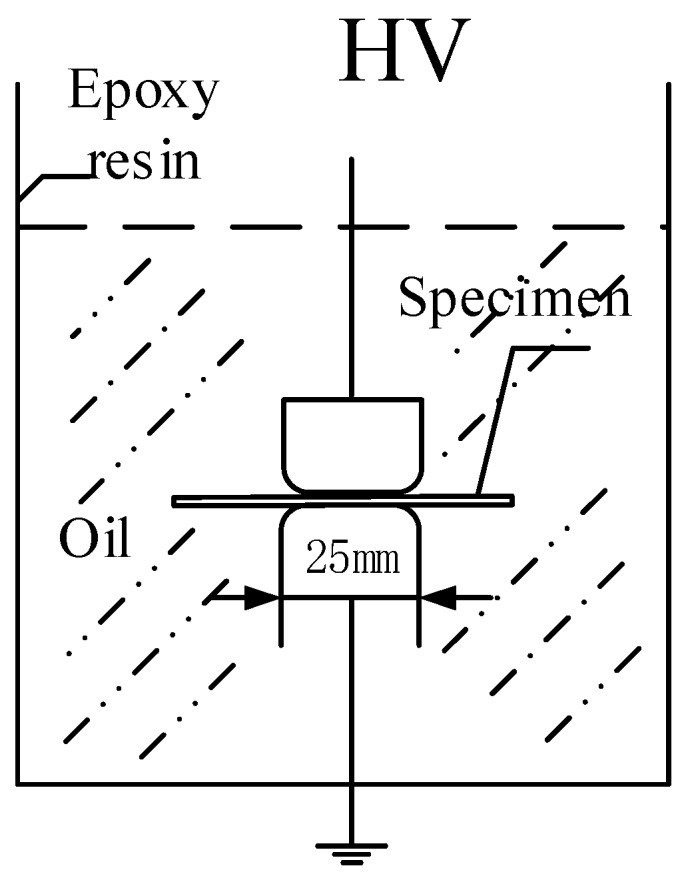
Direct-current (DC) breakdown electric field strength test system.

In addition, high-voltage generators and column polar structure, in compliance with the standard ASTM-D149 were applied during the DC breakdown strength tests, and the entire testing system was placed in transformer oil as shown in Figure 6. The thickness at the breakdown point was measured for calculation. To reduce the influence of data scattering, the average value of multiple measuring data was taken.

**Figure 7:**
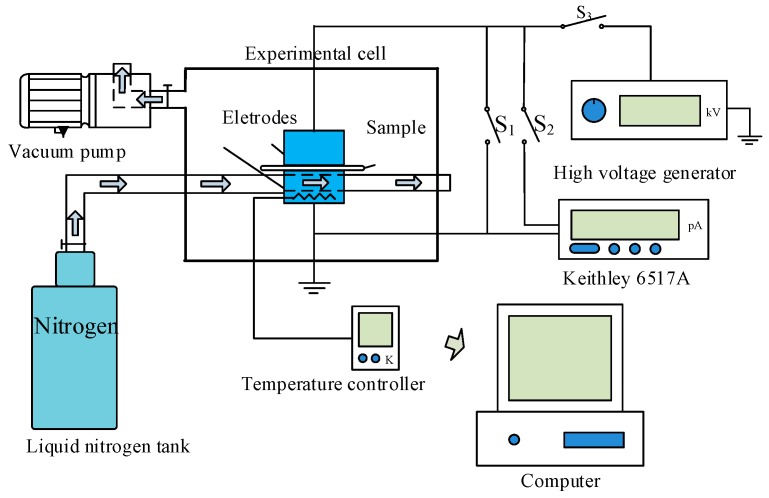
Schematic of thermal simulated current (TSC) measurement system.

**Figure 8:**
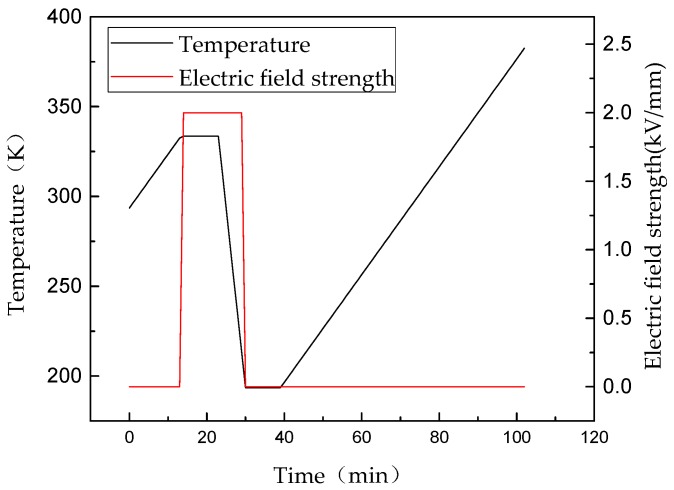
Curves of temperature and electric field stress versus time of TSC test.

To characterize the trap parameters of epoxy non-modified and nano-SiO_2_-modified RIP, the thermally stimulated current (TSC) was tested. The RIP samples were polarized under an electric field of 2 kV/mm at 333 K for 10 min. The temperature was quickly decreased to 193 K using liquid nitrogen. Then, the temperature was linearly increased to 383 K at 3 K/min and the TSC current curve of the sample was measured. The setup of the TSC measurement system and test conditions are shown in Figure 7 and Figure 8, respectively.

## 3. Results

### 3.1. Conductivity Characteristics of Non-Modified and Nano-SiO_2_ Modified RIP

**Figure 9:**
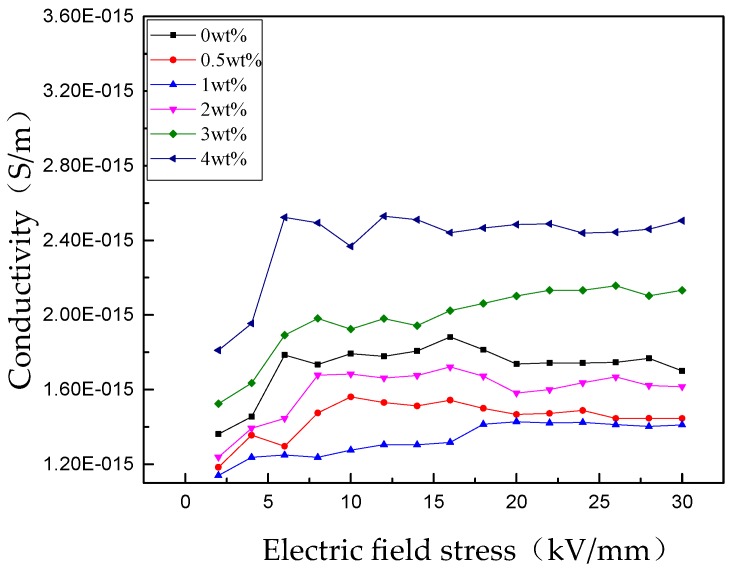
Versus E curves of RIP with different nano-SiO_2_ nanoparticle components.

The relationships between conductivity (*γ*) and electric field stress (*E*) of RIP with different nano-SiO_2_ contents are shown in Figure 9. The conductivities of nano-SiO_2_-modifies RIP are first decreased and then increase with increasing nano-SiO_2_.

### 3.2. Relative Permittivity Characteristics of Non-Modified and Nano-SiO_2_ Modified RIP

**Figure 10:**
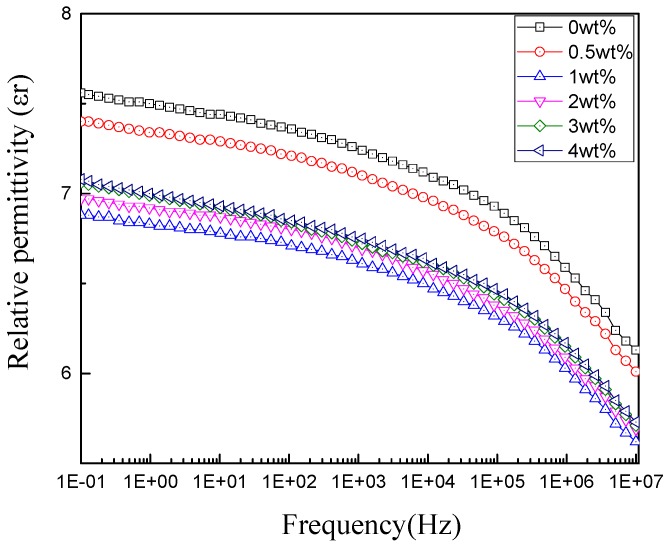
*ε*_r_ versus frequency curves of RIP with different nanoparticle components.

The relationships between relative permittivity (*ε*_r_) and frequency of the non-modified and nano-modified RIP are shown in Figure 10. The *ε*_r_ values of the RIP with the different nano-SiO_2_ content first decrease and then increase with increasing nano-SiO_2_ contents. Furthermore, the *ε*_r_ value of unmodified RIP is greater than that of the nano-SiO_2_ modified RIP. At low frequency area, *ε*_r_ changes slowly with increasing frequency, and decreases rapidly at high frequencies. 

### 3.3. DC Breakdown Strength Characteristics of Non-Modified and Nano-Modified RIP

**Figure 11:**
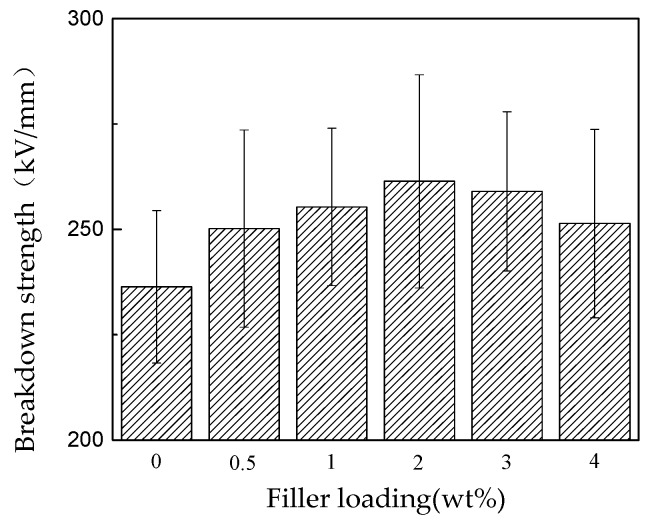
Breakdown strength histogram of RIP with different nanoparticle components.

The relationships between DC breakdown strength and nano-SiO_2_ contents are shown in Figure 11. The electric breakdown strength of nano-SiO_2_-modified RIP rises first increases with increasing content, peaks at 2 wt %, and decreases for 3 and 4 wt %. The maximum DC breakdown strength increased by 10.6% compared to that of no-modified RIP.

### 3.4. TSC Test Results of Epoxy Resin Non-Modified and Nano-SiO_2_ Modified RIP

**Figure 12:**
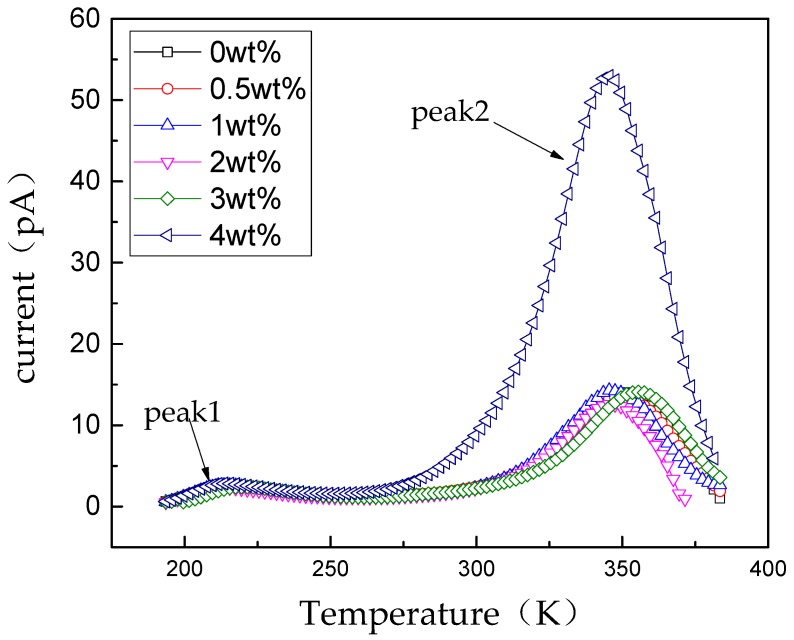
TSC curves of RIP with different nano-SiO_2_ doping components.

For trap parameters of non-modified and nano-SiO_2_-modified RIP are represented by the TSC test results shown in Figure 12. Every current curve has two peaks, one in the low-temperature region at ~213 K and another appears in the high-temperature region at ~350 K. At high temperature, the shape of the current curves firs narrows and then widens with the increasing filler loading. Furthermore, the current curve becomes much wider and higher when the content increase to 4 wt %. The first peak is mainly from the *β* relaxation process [1], while the peak second is closely related to the release of charges from trap states.

## 4. Discussion

**Figure 13:**
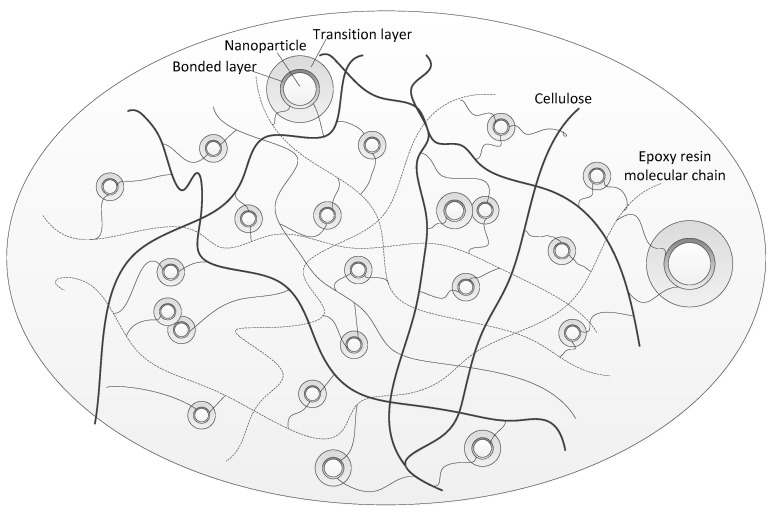
Distribution model of nanoparticles in RIP.

In general, most scholars believe that the dielectric properties of nano-composite dielectrics are closely related to the interface between nanoparticles and polymer matrices [20,21,22]. According to Tanaka’s research, the interface of the nanoparticle can be divided into bonded and transition layers. The bonded layer provides shallow traps, while the transition layer provides deep traps [23]. The distribution of nano-SiO_2_ in the RIP is as shown in Figure 13.

The trap is a localized state in the dielectrics, mainly distributed in the forbidden band gap [24]. Nanoparticles in insulating materials affect the distribution and concentration of traps. Nanoparticles in insulating materials affect the distribution and concentration of traps. Nanoparticle doping can affect matrix traps and introduce more traps with different levels to it [25]. To analyze the effect of nano-SiO_2_ fillers on trap parameters of RIP, the TSDC results from Figure 12 are used. 

The migrated carriers in the sample were easily conducted when the temperature was high and the DC electric field acted on the sample. When the sample temperature decreased rapidly to 193 K, the trapped carriers were “frozen”. The frozen carriers escaped the traps during the subsequent slow warming process via thermal excitation, which formed peak 2. The trapped charge *Q*_TSC_ could be obtained by the following equation:

(1) $$Q_{\mathrm{TSC}}=\int_{t2}^{t1}I(t)dt=\frac{60}{\beta }\int_{T2}^{T1}I(T)dT$$

where *I* (*T*) is the TSC current value, *T*1 and *T*2 are the initial and end temperatures respectively, and *β* is the temperature increase rate (3 K/min).

Meanwhile, the trap level could be calculated according to the half-width method by the following equation:

(2) $$E=\frac{2.47T^{2}{}_{m}k}{\Delta T}$$

where *T*_m_ is the temperature corresponding to the peak current, ∆*T* is the temperature difference between the two half-peak values, and *k* is the Boltzmann constant [23]. The trap parameters of the non-modified and nano-SiO_2_ modified RIP samples are shown in Table 1.

It can be seen that the charge density firstly decreases and then greatly increases with increases in the nano-SiO_2_ loading. The trap level increases first and then decreases with increases in the nano-SiO_2_ loading. The trap density slightly decreases as the trap level becomes deeper. The trap density and trap depth reflect the number of traps and the ability of traps to bind carriers, respectively. 

For the drop of the decrease in the trap density of trap charge density when for the increases in nano-SiO_2_ content (below the 2 wt %) may arise from the lower content of nano-SiO_2_ fixing some localized state in the RIP. However, as the content of nano-SiO_2_ over the 2 wt %, many shallow traps are introduced to the nano-SiO_2_-modified RIP. Thus, it can be seen that the trap density increases greatly while the trap level decreases slightly.

For trap level is the minimum energy necessary for the captured carrier escape the trap. The trap deep increase with increasing (below 2 wt %) may arise from the low loading nano-SiO_2_ forming independent interfaces in the RIP. Independent interfaces in the RIP have strong physical and chemical effects, which can deepen trap levels. With continued increases in nano-SiO_2_ content, the distance between nano-SiO_2_ particles is decreased sharply, and some of the interfaces overlap. The physical and chemical effects become weaker, the transition layer becomes wider, and the traps become shallower.

For the relative permittivity, nano-SiO_2_ itself is non-polar with a small relative permittivity, which decreases the relative permittivity of nano-modified RIP to less than that of the non-modified RIP. However, the low-content nano-SiO_2_ can form the independent interfaces with strong physical and chemical effects and prevent the end of the molecular chain movement in RIP. With higher content of nano-SiO_2_ in the RIP, the interfaces overlap and the interfacial effects are weakened. Therefore, the relative permittivity of nano-SiO_2_-modified RIP is reduced overall. As a result, it decreases at first and then increases with increasing nano-SiO_2_ loading.

**Figure 14:**
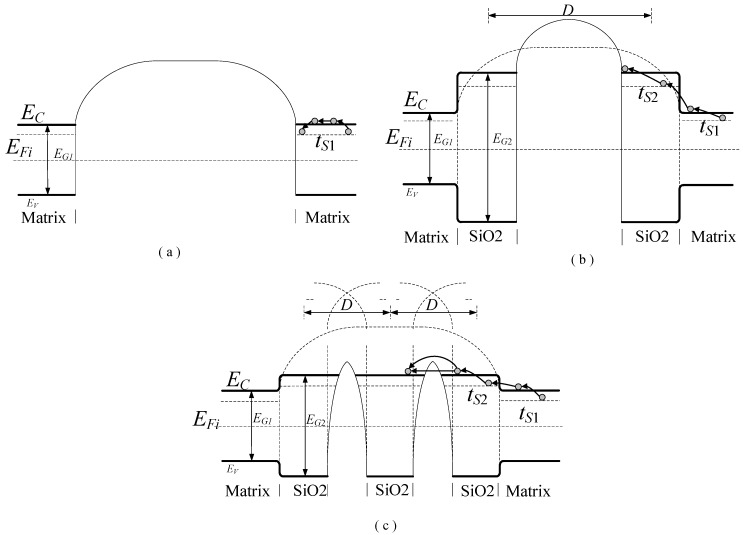
(**a**) band structure of epoxy resin impregnated non-modified pressboard; (**b**)Energy band structure of epoxy resin impregnated modified pressboard with low content SiO_2_; (**c**)Energy band structure of epoxy resin impregnated modified pressboard high content SiO_2._

Furthermore, the nano-SiO_2_ filling of the RIP affects the energy band structure of RIP. Based on our previous research, a simulated energy band structure for nano-modified pressboard is shown in Figure 14 [19]. The forbidden band of the RIP is wide and high, as shown in Figure 14a, which causes the low conductivity of non-modified RIP. A small amount of nano-SiO_2_ filling in RIP can cause the forbidden band to become wider and higher, as shown in Figure 14b, which can hinder carrier transport. Furthermore, when the nano-SiO_2_ content reaches a certain value, the distance between nano-SiO_2_ particles decreased, the interfacial effects are weakened, and the carriers can jump between nano-SiO_2_ as shown in Figure 14c. The transmission paths of carriers are increased. This shows that the conductivity of nano-SiO_2_-modified RIP firstly decreases and then increases with increasing nano-SiO_2_ contents.

In which *D* is the separation distance between neighbor nanoparticles, *E*_C_ is conduction band, *E*_V_ is the valence band, *E*_Fi_ is the Fermi level, *E*_G1_ is the width of the forbidden band of epoxy impregnated pressboard (matrix), and *E*_G2_ is the width of the forbidden band of SiO_2_ nanoparticles, while *t*_s1_ and *t*_s2_ are the trap energy levels of the matrix and SiO_2_ nanoparticles, respectively. According to Figure 12, the transition energy rises at first and falls with increasing nano-SiO_2_ content.

For the increase of the DC breakdown strength of the nano-SiO_2_ RIP, some studies indicated that breakdown was closely related to the process of carrier transport. First, the nano-SiO_2_ particles in the RIP can “scatter” the carriers, decreasing the energy and the free average path of the carriers [25]. Second, the nano-SiO_2_ occupies some free volume in the RIP, which decreases the free average path of the carriers. Therefore, the carriers receive insufficient energy from the electric field for transport. In addition, the copolar space charge near the electrode may decrease the electric field applied to the sample [26]. These above reasons can explain appropriately why the nano-SiO_2_ doping increases the DC breakdown strength.

**Figure 15:**
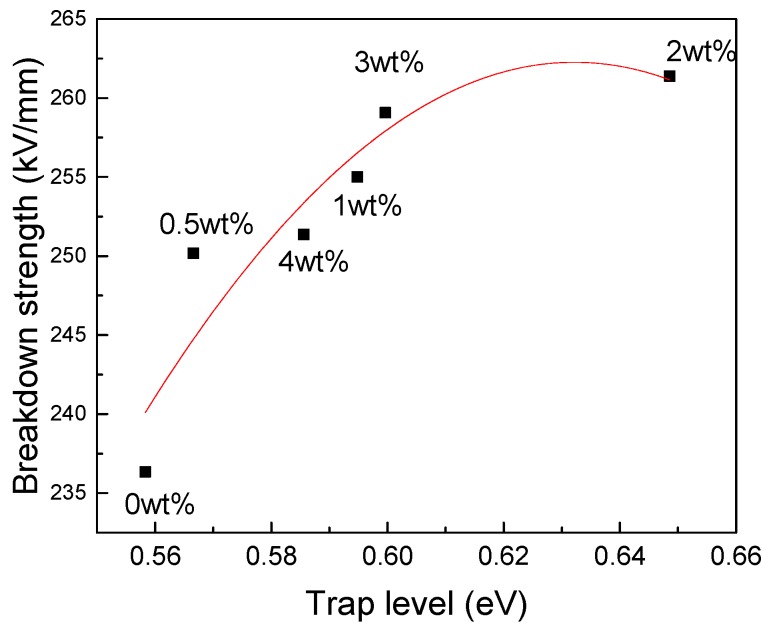
Relationship between breakdown strength and trap level.

The relationship found in this study between the trap level and the DC breakdown strength is shown in Figure 15. It shows that the DC breakdown strength increases with the trap level. The change of the trap level mainly affects the process of carrier transport in an electric field. The carriers entering and exiting the trap consume significant energy; deeper traps correspond to greater energy consumption.t The deeper traps impede effective carrier formation, and most energy is consumed when the carriers escape the traps., The low-energy carriers hit the molecular chains in the RIP matrix, causing less damage to the molecular chains. For this reason, the DC breakdown strength increases with increasing of trap level. Changes in the trap parameters have a major impact on the dielectric properties of nano-SiO_2_-modified RIP.

## 5. Conclusions

Based on the experimental study on the dielectric properties of nano-SiO_2_ modified RIP, the following conclusions have been drawn:

1. The nano-SiO_2_ particles can alter the trap depth and density; within a specific range, the trap depth increased and the trap is decreased.
2. The trap depth is closely related to the DC breakdown strength, and the DC breakdown strength increased with increasing of the trap depth.
3. Nano-SiO_2_ doping at an appropriate content can increase the DC breakdown strength, while simultaneously decreasing the volume conductivity and relative permittivity of the RIP.

## Figures and Tables

**Table 1:** Trap parameters of RIP with different nanoparticle component.

Nanoparticle Components	Peak Current Value (pA)	Peak Value Temperature (K)	Trap Charge Quantity (nC)	Trap Level (eV)
non-modified	10.7	349	17.1	0.5583
0.5 wt % SiO_2_	13.5	349	16.91	0.5666
1 wt % SiO_2_	14.3	345	16.67	0.5948
2 wt % SiO_2_	12.6	345	13.46	0.6486
3 wt % SiO_2_	14.1	349	17.4	0.5996
4 wt % SiO_2_	53	345	54.2	0.5856

## References

[1] Ning X., Peng Z., Feng H., Liu P. (2015). Dielectric properties of epoxy and epoxy/creep paper composites for UHVDC dry casings. Chin. J. Electr. Eng..

[2] Peng L.I., Hai-Yun J.I., Hui-Cheng S.H., Nai-Kui G.A., Zhong-Ren P.E. (2009). Investigation on dielectric properties of epoxy/crepe paper composites for ultra-high voltage DC bushing. High Voltage App..

[3] Ning X., Feng H., Zhang H., Liu P., Xiang Z., Peng Z. (2015). Dielectric properties of multi-layer epoxy resinimpregnated crepe paper composites. IEEE Trans. Dielectr. Electr. Insul..

[4] Krivda A., Tanaka T., Frechette M., Castellon J., Fabiani D., Montanari G.C., Gorur R., Morshuis P., Gubanski S., Kindersberger J. (2017). Characterization of epoxy microcomposite and nanocomposite materials for power engineering applications. IEEE Electr. Insul. Mag..

[5] Preetha P., Thomas M.J. (2011). Partial discharge resistant characteristics of epoxy nanocomposites. IEEE Trans. Dielectr. Electr. Insul..

[6] Das S., Gupta N. (2010). Study of space charge characteristics in epoxy resin and its nanocomposites. Proceedings of the 2010 10th IEEE International Conference on Solid Dielectrics.

[7] Katayama J., Ohki Y., Fuse N., Kozako M., Tanaka T. (2013). Effects of nanofiller materials on the dielectric properties of epoxy nanocomposites. IEEE Trans. Dielectr. Electr. Insul..

[8] Song G.S., Lee D.S., Kang I. (2016). The Effects of in Situ-Formed Silver Nanoparticles on the Electrical Properties of Epoxy Resin Filled with Silver Nanowires. Polymers.

[9] Nezhad H.Y., Thakur V.K. (2018). Effect of morphological changes due to increasing carbon nanoparticles content on the quasi-static mechanical response of epoxy resin. Polymers.

[10] Hanemann T., Szabó D.V. (2010). Polymer-Nanoparticle Composites: From Synthesis to Modern Applications. Materials.

[11] Kamata Y., Ohe E., Endoh K., Furukawa S., Tsukioka H., Masejima M., Fujita H., Nozaki M., Ishizuka F., Hyohdoh K. (1991). Development of low-permittivity pressboard and its evaluation for insulation of oil-immersed EHV power transformers. IEEE. Trans. Dielectr. Electr. Insul..

[12] Tang W.W., Zeng G.M., Gong J.L., Liu Y., Wang X.Y., Liu Y.Y., Liu Z.F., Chen L., Zhang X.R., Tu D.Z. (2012). Simultaneous adsorption of atrazine and Cu (II) from wastewater by magnetic multi-walled carbon nanotube. Chem. Eng. J..

[13] Liao R.J., Yuan L., Zhang F.Z., Yang L.J., Wang K., Duan L. (2014). Preparation of montmorillonite modified insulation paper and study on its electrical characteristics. High Volt. Eng..

[14] Liao R.J., Lv C., Wu W.Q., Liu T. (2014). Insulating property of insulation paper modified by Nano-TiO_2_. High Volt. Eng..

[15] Bai G., Liao R.J., Liu N., Liu H.B., Yang L.J., Shakeel A. (2015). Influence of Nano-AlN Modification on the Dielectric Properties of Meta-aramid Paper. High Volt. Eng..

[16] Yang Y., Zhang J., Zhou C., Wu S., Xu S., Liu W., Han H., Chen B., Zhao X.Z. (2008). Effect of lithium iodide addition on poly(ethylene oxide)-poly(vinylidene fluoride) polymer-blend electrolyte for dye-sensitized nanocrystalline solar cell. J. Phys. Chem. B.

[17] Liao R.J., Lv C., Yang L.J., Zhang Y.Y., Liu T. (2013). Space Charge Behavior in Oil-Impregnated Insulation Paper Reinforced with Nano-TiO_2_. Bioresources.

[18] Chen Q.G., Liu H.Q., Zhuge X.L., Wei X.L. (2014). Analysis of dielectric properties and electric field homogenization of modified insulation pressboard based on nano SiC. Electr. Mach. Control.

[19] Chen Q., Liu H., Chi M., Wang Y., Wei X. (2017). Experimental Study on Influence of Trap Parameters on Dielectric Characteristics of Nano-Modified Insulation Pressboard. Materials.

[20] Wang X., Nelson J.K., Schadler L.S., Hillborg H. (2010). Mechanisms leading to nonlinear electrical response of a nano p-sic/silicone rubber composite. IEEE Trans. Dielectr. Electr. Insul..

[21] Green M.L., Rhine W.E., Calvert P., Bowen H.K. (1993). Preparation of poly(ethylene glycol)-grafted alumina. J. Mater. Sci. Lett..

[22] Tu Y.P., He J., Wang Q., Liu M., Xu G.L., Ding L.J. (2010). Measurement of thermally stimulated current in ZnO varistor. Proc. CSEE.

[23] Li S., Yin G., Chen G., Li J., Bai S., Zhong L., Zhang Y., Lei Q. (2010). Short-term breakdown and long-term failure in nanodielectrics: A review. IEEE Trans. Dielectr. Electr. Insul..

[24] Dissado L.A., Fothergill J.C. (1992). Electrical Degradation and Breakdown in Polymers.

[25] Tanaka T. (2005). Dielectric nanocomposites with insulating properties. IEEE Trans. Dielectr. Electr. Insul..

[26] Roy M., Nelson J.K., Maccrone R.K., Schadler L.S. (2007). Candidate mechanisms controlling the electrical characteristics of silica/xlpe nanodielectrics. J. Mater. Sci..

